# Benefit of Physiologically Variable Over Pressure-Controlled Ventilation in a Model of Chronic Obstructive Pulmonary Disease: A Randomized Study

**DOI:** 10.3389/fphys.2020.625777

**Published:** 2021-01-13

**Authors:** Andre Dos Santos Rocha, Roberta Südy, Davide Bizzotto, Miklos Kassai, Tania Carvalho, Raffaele L. Dellacà, Ferenc Peták, Walid Habre

**Affiliations:** ^1^Unit for Anaesthesiological Investigations, Department of Acute Medicine, University Hospitals of Geneva, University of Geneva, Geneva, Switzerland; ^2^Dipartimento di Elettronica, Informazione e Bioingegneria, Politecnico di Milano, Milan, Italy; ^3^Instituto de Medicina Molecular, Faculdade de Medicina, Universidade de Lisboa, Lisbon, Portugal; ^4^Department of Medical Physics and Informatics, Faculty of Medicine, University of Szeged, Szeged, Hungary

**Keywords:** COPD, variable ventilation, animal model, gas exchange, lung mechanics

## Abstract

**Introduction:**

The advantages of physiologically variable ventilation (PVV) based on a spontaneous breathing pattern have been demonstrated in several respiratory conditions. However, its potential benefits in chronic obstructive pulmonary disease (COPD) have not yet been characterized. We used an experimental model of COPD to compare respiratory function outcomes after 6 h of PVV versus conventional pressure-controlled ventilation (PCV).

**Materials and Methods:**

Rabbits received nebulized elastase and lipopolysaccharide throughout 4 weeks. After 30 days, animals were anesthetized, tracheotomized, and randomized to receive 6 h of physiologically variable (*n* = 8) or conventional PCV (*n* = 7). Blood gases, respiratory mechanics, and chest fluoroscopy were assessed hourly.

**Results:**

After 6 h of ventilation, animals receiving variable ventilation demonstrated significantly higher oxygenation index (PaO_2_/FiO_2_ 441 ± 37 (mean ± standard deviation) *versus* 354 ± 61 mmHg, *p* < 0.001) and lower respiratory elastance (359 ± 36 *versus* 463 ± 81 cmH_2_O/L, *p* < 0.01) than animals receiving PCV. Animals ventilated with the variable mode also presented less lung derecruitment (decrease in lung aerated area, –3.4 ± 9.9 *versus* –17.9 ± 6.7%, *p* < 0.01) and intrapulmonary shunt fraction (9.6 ± 4.1 *versus* 17.0 ± 5.8%, *p* < 0.01).

**Conclusion:**

PVV applied to a model of COPD improved oxygenation, respiratory mechanics, lung aeration, and intrapulmonary shunt fraction compared to conventional ventilation. A reduction in alveolar derecruitment and lung tissue stress leading to better aeration and gas exchange may explain the benefits of PVV.

## Introduction

Chronic obstructive pulmonary disease (COPD) is the most prevalent respiratory disease, with a reported prevalence of 251 million cases worldwide (Chronic obstructive pulmonary disease (COPD), 2017). In addition to the high burden and morbidity of the disease (Gershon et al., 2013; Lopez-Campos et al., 2016), COPD is the third leading cause of death worldwide (Lozano et al., 2012). COPD is characterized by chronic lung inflammation with airway remodeling and airflow limitation, which is associated with irreversible emphysematous destruction of the alveoli. These permanent alterations of the pulmonary structure progressively impair gas exchange and respiratory mechanics, leading to different degrees of respiratory insufficiency.

Chronic obstructive pulmonary disease is a progressive disease with exacerbations leading potentially to acute respiratory failure necessitating mechanical ventilation (Gacouin et al., 2015). Furthermore, even under stable conditions, COPD patients may require mechanical ventilation for respiratory life-support while undergoing general anesthesia. Considering that COPD patients have a higher incidence of respiratory complications induced by mechanical ventilation (Edrich and Sadovnikoff, 2010; Hausman Jr., Jewell and Engoren, 2015; Numata et al., 2018; Szylinska et al., 2020), it is therefore of utmost importance to optimize ventilation modalities in this population.

Variable ventilation is a recently developed modality that mimics physiological breathing by incorporating breath-by-breath variations in tidal volume and respiratory rate. A variable breathing pattern has been advocated to be superior to monotonous ventilation by means of optimizing gas exchange and recruitment of alveoli, given the non-linearity of the respiratory system (Suki et al., 1998; Brewster et al., 2005). Several studies have demonstrated the benefits of variable ventilation on gas exchange (Carvalho et al., 2009, 2011) and lung mechanics as well as in preventing ventilator-induced lung injury in animal models with normal lungs (Mutch et al., 2000a; Arold et al., 2003; Walesa et al., 2018), acute respiratory distress syndrome (ARDS) (Lefevre et al., 1996; Boker et al., 2002; Funk et al., 2004; Spieth et al., 2009), emphysema (Henriques et al., 2016), and prematurity (Pillow et al., 2011; Berry et al., 2012) and in the presence of atelectasis (Mutch et al., 2000b; McMullen et al., 2006) and asthma (Dos Santos Rocha et al., 2020). However, the potential beneficial effects of variable ventilation in a model of COPD have not yet been characterized. We hypothesize that the deterioration of lung mechanics and oxygenation during mechanical ventilation of lungs with main features of COPD will be prevented by a ventilation mode reproducing the variability of spontaneous breathing. To test this hypothesis, we compared physiologically variable ventilation (PVV) to conventional pressure-controlled ventilation (PCV) in an experimental model of COPD.

## Materials and Methods

### Ethics Statement

The current study was approved by the Animal Welfare Committee of the Canton of Geneva and the Experimental Ethics Committee of the University of Geneva, Switzerland (GE 184/18, 2 January 2019). All procedures were performed in accordance to current Swiss animal protection laws (LPA, RS455). The ARRIVE guidelines were followed to report this study.

### Study Design

**FIGURE 1:**
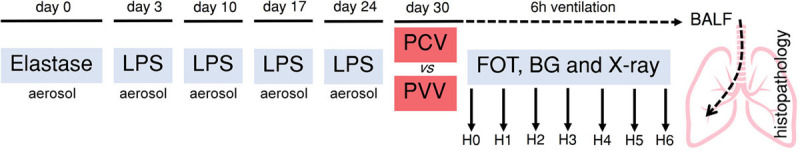
Schematic representation of the experimental protocol. LPS, lipopolysaccharide; PCV, pressure-controlled ventilation; PVV, physiologically variable ventilation; FOT, forced oscillation technique; BG, blood gas; BALF, bronchoalveolar lavage fluid; H0 to H6, measurement time points at hour 0 to hour 6.

The study protocol is represented in Figure 1. Adult New Zealand White rabbits [male *n* = 8, female *n* = 7, aged 20 weeks, weighing 3.4 kg (range 2.88–3.76 kg)] were purchased from the University of Geneva’s farm (Arare, Geneva, Switzerland). Pathological aspects of COPD were experimentally induced over 4 weeks. On day 30, rabbits were anesthetized, tracheotomized, and randomized to receive 6 h of either PCV or PVV. After 6 h, animals were euthanized with sodium thiopental (100 mg/kg), and lung post-mortem analyses were performed.

### Experimental Procedures

#### COPD Model Preparation

All rabbits received a scheme of aerosol treatments over 4 weeks, as described in Figure 1, to induce a persistent lung injury that reproduced pathological aspects of COPD, as described previously (Sajjan et al., 2009; Ganesan et al., 2010). On day 0, porcine elastase 15 U/kg (Elastase suspension 3 U/mg Worthington, BioConcept, Allschwil, Switzerland) was aerosolized using a vibrating mesh nebulizer (Aerogen^®^ Solo Nebulizer System, Hamilton Medical, Switzerland). Subsequently, once a week from day 3 to day 24, rabbits received nebulized lipopolysaccharide 20 μg/kg (*Escherichia coli* O111:B4, Sigma, St. Louis, MO, United States). All nebulized substances were delivered to the lower airways through a supraglottic airway device (v-gel^®^, Docsinnovent Ltd., London, United Kingdom), to reduce the mucosal damage of repeated intubation (Engbers et al., 2017) and aerosol dispersion in the upper airway. Under sedation with 2% sevoflurane for approximately 10 min (the duration of the nebulization), animals received pressure-support ventilation with 10 cmH_2_O of inspiratory pressure and 3 cmH_2_O of positive end-expiratory pressure (PEEP) using a clinical ventilator (Primus^®^, Dräger, Lübeck, Germany). After elimination of sevoflurane, animals were weaned from the ventilator and the supraglottic airway device was removed. Periprocedural care included artificial tears, external heating, and supplemental oxygen. Furthermore, an animal welfare score was quantified after each nebulization and twice per week, to assess the general and respiratory condition of the rabbits (Supplementary Table S1). In case of respiratory distress, supplemental oxygen was administered until symptoms resolved. The rabbits had access to food and water *ad libitum* before and after the experiments.

#### Anesthesia and Surgical Preparation

On day 30, anesthesia was induced by intramuscular injection of ketamine 25 mg/kg and xylazine 3 mg/kg. Cannulation of the ear vein with a 24 G catheter (Abbocath, Abbott Medical, Baar/Zug, Switzerland) was performed. After infiltration of the anterior cervical region with lidocaine 1% (Sintetica, Mendrisio, Switzerland), a surgical tracheostomy with a 3.5-mm uncuffed tube (3.5 mm Portex, Smiths Medical, Kent, United Kingdom) was performed. Intravenous anesthesia with propofol 10 mg/kg/h, fentanyl 5 μg/kg/h, and midazolam 0.2 mg/kg/h was administered via the ear vein. The left femoral artery and right internal jugular vein were cannulated with a 20 G catheter for arterial and venous blood sampling and invasive blood pressure measurements.

After confirming adequate anesthesia and analgesia through the absence of movement in response to painful stimuli and cardiovascular monitoring (stable heart rate and arterial blood pressure), neuromuscular blockade was performed with atracurium besylate 0.6 mg/kg/h. Body temperature was monitored with a rectal thermometer and kept between 38 and 39°C with a thermostatic heating pad (Harvard Apparatus, South Natick, MA, United States). Intravenous fluid replacement was administered with Ringer’s acetate 2 mL/kg/h.

#### Mechanical Ventilation Settings

Mechanical ventilation was applied using a computer-controlled custom-made turbine ventilator connected to a heated pediatric pneumotachograph (PNT 3700 Hans Rudolph Inc., Shawnee, KS, United States) and pressure transducers (Honeywell Differential Pressure Sensor model 24PCEFA6D, Charlotte, NC, United States). Custom-made software designed in Labview^®^ was used to control the ventilator and to continuously record tracheal airflow (V′), airway pressure (P_aw_), and tidal volume (V_*T*_). After tracheostomy and surgical preparation, a sustained inspiratory pressure of 25 cmH_2_O was applied twice for 10 s to normalize lung volume history in all animals. Subsequently, animals were randomized to receive 6 h of either PCV or PVV. While the ventilatory pattern was essentially different between PCV and PVV, all the ventilation parameters were set equally between groups, as follows: an inspiratory pressure was set to deliver an average V_*T*_ of 7 mL/kg, a PEEP of 3 cmH_2_O, a fraction of inspired oxygen (FiO_2_) of 0.4, and an inspiratory to expiratory ratio of 1:3. The driving pressure (P_*driving*_, calculated as the difference between PEEP and peak inspiratory pressure), set at H0 to deliver the target V_*T*_, was kept constant throughout the 6 h of ventilation. Mainstream capnography was used throughout the experiment and respiratory rate was adapted to achieve normocapnia (end-tidal CO_2_ of 5.5–6%).

**FIGURE 2:**
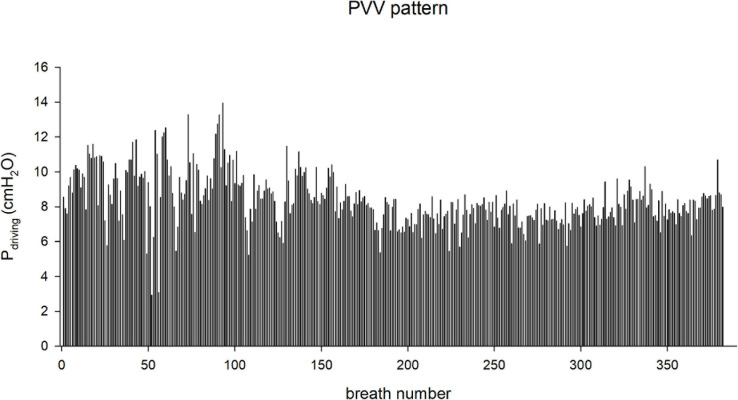
Changes in the driving pressure (P_*driving*_) during the application of physiological variable ventilation (PVV) pattern, in a representative rabbit.

While the PCV consisted in a monotonous pattern, as per conventional use in clinical practice, the PVV pattern, whose characteristics are summarized in Figure 2 and Table 1, reproduced the (physiologically variable) breathing pattern in awake COPD rabbits. To obtain the PVV pattern, whole-body plethysmography was performed in a subgroup of four animals, between experimental days 27 and 30 to repeatedly record their spontaneous breathing after COPD induction. Briefly, a pressure transducer (Honeywell Differential Pressure Sensor model 24PCEFA6D, Charlotte, NC, United States) was connected to a custom-made plexiglass box (external measures of 300 × 300 × 500 mm, internal volume of 37.63 L) and the pressure signal was digitized at 1 kHz (ADInstruments, Powerlab model 8/35 and LabChart 7, Dunedin, New Zealand) with the simultaneous monitoring of movements by a digital camera. The box temperature and humidity were recorded and kept constant with a fresh air supply of 1.5 L/min. Rabbits were placed in the plethysmograph box for 30-min periods for four consecutive days to get accustomed to the handling and the box environment. Ten-minute recordings of spontaneous breathing on the fourth day, when animals were accustomed to the box environment, were used to produce the PVV pattern. Recording segments corresponding to movement artifacts were removed. Replicates of the recorded pattern were created with different average respiratory rates, maintaining the exact ratios of breath-to-breath pressure and frequency for each replicate. The resulting PVV pattern file contained 382 breaths that were reproduced in loop for the duration of the experimental protocol.

**TABLE 1:** Characteristics of the ventilation variables, tidal volume (V_*T*_), and respiratory rate (RR) for pressure-controlled ventilation (PCV) and PVV during the first hour of ventilation.

	**PCV**		**PVV**	
	**V_*T*_ (mL/kg)**	**RR (1/min)**	**V_*T*_ (mL/kg)**	**RR (1/min)**
Mean	7.20.7	24.21.4	7.10.6	22.91.5
CV (%)	1.90.8	0.20.1	12.61.0	12.90.4
Minimum	6.80.6	24.11.4	3.70.4	13.10.9
Maximum	7.40.8	24.31.4	10.70.7	35.52.5

*CV, coefficient of variation.*

#### Measurement of Respiratory Mechanics

The impedance spectra of respiratory system (Z_*rs*_) were measured using forced oscillatory technique, as described in detail previously (Hantos et al., 1992; Peták et al., 2006; Albu et al., 2018). Briefly, 2 cmH_2_O peak-to-peak amplitude pseudorandom oscillations (15 non-integer multiples between 0.5 and 21 Hz) were applied for 10 s during end-expiratory pauses by the computer-controlled ventilator turbine. The V′ was measured using a pneumotachograph (PNT 3700 Hans Rudolph Inc., Shawnee, KS, United States) connected to a differential pressure transducer (Honeywell model 24PCEFA6D, Charlotte, NC, United States). A second pressure transducer connected to a side port of the tracheal cannula was used to measure P_*aw*_. Z_*rs*_ (Z_*rs*_ = P_*aw*_/V′) was calculated using Fast Fourier Transformation from the 10-s-long recordings with 4-s time windows and 95% overlap.

Three epochs were recorded and averaged in each measurement timepoint. The impedance of the breathing circuit was subtracted from measured impedance spectra. To separate the airway and respiratory tissue mechanical properties, we fitted a well-validated model (Hantos et al., 1992) to the measured Z_*rs*_ spectra. The model contained airway resistance (R_*aw*_) and inertance (I_*aw*_), in series with a tissue model including damping (G) and elastance (H). A global optimization procedure was used to minimize the differences between the measured and modeled impedance values.

As previously described (Petak et al., 1997), R_*aw*_ and I_*aw*_ reflect the flow resistance and mass inertia of the intrapulmonary gas, respectively. The tissue parameters G and H characterize the energy loss (viscous resistance) and storage (elastance) in the respiratory tissues, respectively.

#### Measurement of Blood Parameters

Arterial and venous blood was analyzed by a point-of-care blood gas analyzer (i-Stat, Abbott Laboratories, Chicago, IL, United States). Partial pressure of oxygen (PaO_2_) and carbon dioxide (PaCO_2_) were assessed. Oxygenation index was calculated as PaO_2_/FiO_2_. Intrapulmonary shunt fraction (Qs/Qt) was determined as the ratio of pulmonary end-capillary oxygen content (CcO_2_) minus arterial oxygen content (CaO_2_), divided by the CcO_2_ minus the central venous oxygen content (CvO_2_).

$$\frac{{\text{Q}}_{\text{s}}}{{\text{Q}}_{\text{t}}}=\frac{{\text{CcO}}_{2}-{\text{CaO}}_{2}}{{\text{CcO}}_{2}-{\text{CvO}}_{2}}$$

The total and differential white blood cell counts in the plasma were obtained from arterial blood using a pocH-100iV DIFF hematology analyzer (Sysmex Digitana AG, Horgen, Switzerland).

#### Quantification of Lung Aeration

Structural imaging of the respiratory system was acquired hourly using X-ray fluoroscopic technology (Ziehm Vista C-Arm System, Nuremberg, Germany). The position and distance of the X-ray beam generator and detector were constant during the 6-h ventilation. For each recording, a frame in end-expiration was used for manual segmentation of the lung, based on radiodensity, using pixel counting tool with a custom-made script in MATLAB^®^. Lung aeration area was calculated in pixels from the segmented two-dimensional images image by a radiologist blinded to group allocations and compared in percentage change to the reference image at H0.

#### Lung Histological, Cellular, and Protein Assessment

For morphological and morphometric evaluation, the left lung was fixed by infusing 10% neutral-buffered formalin into the cannulated main bronchus at a hydrostatic pressure of 20 cmH_2_O, followed by immersing in a container with the same fixative for > 48 h. The organ was then embedded in agarose, and the tissue was cut into 18 step slices, anterior to posterior, spaced by 3 mm and processed for paraffin-embedding (six from the cranial lobe and 16 from the caudal lobe). Sections (4 μm) were stained with hematoxylin-eosin, digitally scanned in Nanozoomer SQ (Hamamatsu), and visualized in NDP2.view software. Morphological analysis was performed by a pathologist blinded to group allocations and scored following American Thoracic Society guidelines (Matute-Bello et al., 2011). The scoring system included the presence of inflammatory cells in the alveolar and interstitial spaces, hyaline membranes, proteinaceous debris filling the airspaces, and alveolar septal thickening. Overall lung injury score was calculated by averaging the score for each of the 18 lung slices.

Score for emphysema was performed (i) by a pathologist using a five-tier system with a grading scale (0, absent; 1, minimal; 2, mild; 3, moderate; 4, marked), in which classification is based on the most severe lesions, and (ii) by automated measurement of airspace size, performed using mean linear intercept (MLI) method, as previously described (Crowley et al., 2019). The average MLI was calculated from all 18 microscopy fields at 10x magnification, avoiding big vessels and airways, and with the same cranio-caudal distribution of the histological injury score described above. Lung tissue from naive New Zealand White rabbits with comparable age and weight to the current study animals was used as control (*n* = 3).

The cannulated main bronchus of the right lung was flushed with 20 mL warm phosphate buffered saline containing 1% bovine serum albumin, to obtain bronchoalveolar lavage fluid (BALF). Subsequently, the right lung was stored at –80°C, and lung tissue homogenate was obtained by sonication. The BALF was centrifuged at 412 g for 5 min at 5°C and the supernatant stored at –20°C until analysis. The total and differential cell counts in the BALF were analyzed as described in previous work (Walesa et al., 2018). Enzyme-linked immunosorbent assays were performed according to the manufacturer’s instructions using frozen lung tissue homogenates and undiluted supernatant of the BALF to quantify the inflammatory cytokines interleukin (IL)-6 and IL-8 (Raybiotech Norcross, GA, United States), tumor necrosis factor (TNF-α, MyBiosource MBS2021700, San Diego, CA, United States), surfactant protein B (SP-B, LSBio LS-F47557, Muttenz, Switzerland), surfactant protein D (SP-D, Blue Gene Biotech E04S0170, Paris, France), and E-cadherin (LSBio LS-F43438, Muttenz, Switzerland).

### Study Outcomes

The primary outcomes of the present study were the PaO_2_/FiO_2_ and H after 6 h of ventilation. Secondary outcomes included mean V_*T*_, Qs/Qt, lung aeration area, mechanical parameters R_*aw*_ and G, lung histological injury score, plasma and BALF cell counts, and cytokine levels.

### Statistical Methods

Data are presented as mean ± standard deviation. Normality of each variable distribution was assessed using the Shapiro–Wilk test. Two-way repeated measures analyses of variances were used to analyze the absolute values of primary and secondary outcomes, using ventilation mode (PCV or PVV) and time (H0 to H6) as between and within subject factors, respectively. In case of significance, Dunnett’s *post hoc* test was used to assess significances for ventilation mode (using PCV as reference) and time (using H0 as a reference). Relative changes between H0 and H6 were analyzed using a paired *t*-test or a Mann–Whitney test, depending on normality. Correlation between H and PaO_2_/FiO_2_ was analyzed using Pearson’s correlation test. All statistical tests were performed using SigmaPlot (Version 13, Systat Software, Inc., Chicago, IL, United States). Results were considered significant for a level of *p* < 0.05, and all *p*-values are two-sided.

#### Sample Size Estimation

The sample size was estimated based on respiratory tissue elastance (H), using data previously obtained under similar conditions in rabbits (Walesa et al., 2018). We aimed at detecting 20% differences between groups, assuming an interindividual variation of 15%, a statistical power of 0.8, and a two-sided alpha error of 0.05. The calculation resulted in a minimum sample size of 10 rabbits per group. Considering a potential 10% drop-out rate, we induced experimental COPD on 22 rabbits.

## Results

### Study Population

Chronic obstructive pulmonary disease induction was performed on 22 rabbits. Seven animals were not included in the 6-h ventilation protocol due to lethal alveolar hemorrhage at day 0 after the elastase nebulization. Accordingly, 15 rabbits were randomized at day 30 to receive PCV (*n* = 7) or PVV (*n* = 8) (Figure 1).

### Ventilation Parameters

**FIGURE 3:**
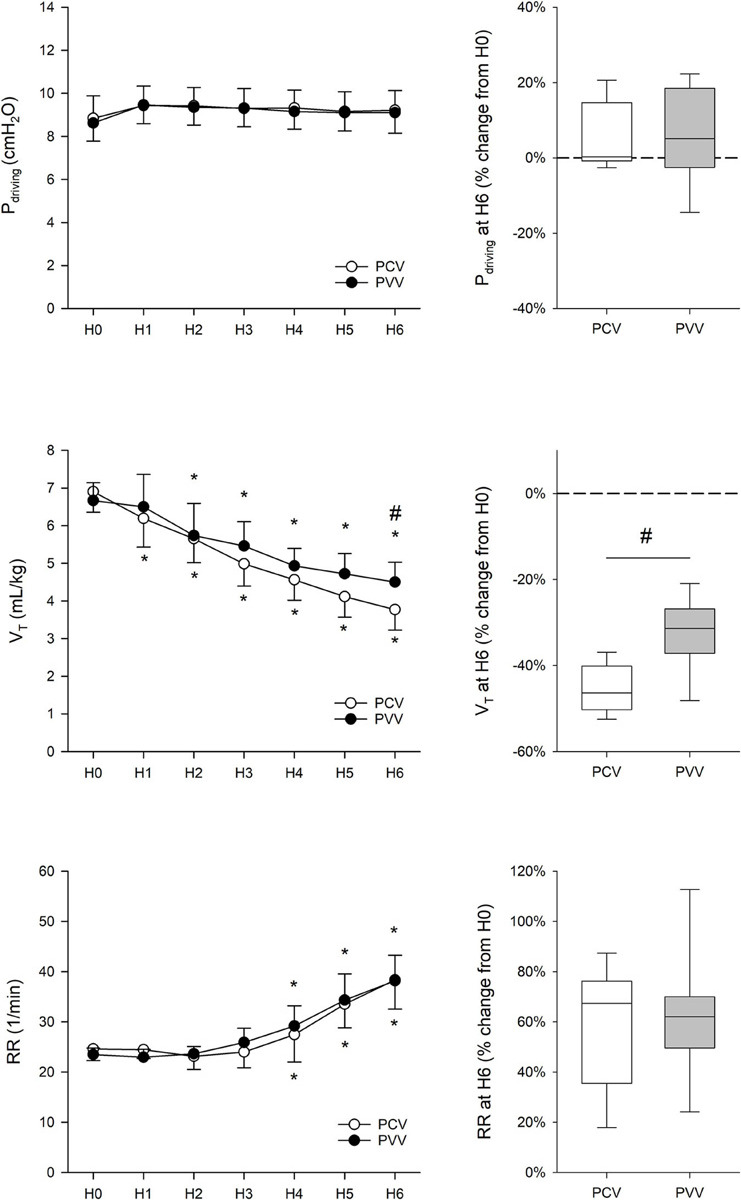
Driving pressure (P_*driving*_), tidal volume (V_*T*_), and respiratory rate (RR), measured at the onset (H0) and throughout the 6 h of ventilation (H1 to H6). Results from physiologically variable ventilation (PVV, filled circles) and pressure-controlled ventilation (PCV, empty circles) are expressed as mean ± standard deviation. Relative changes compared to H0 after the application of 6-h PCV (white box) or PVV (gray box) are reported on the right panels. *p < 0.05 *versus* H0; #*p* < 0.05 *versus* PCV.

The ventilatory parameters are summarized in Figure 3. Mean inspiratory pressure, PEEP, driving pressure, and FiO_2_ were maintained unchanged during the 6-h ventilation with no difference in these parameters between the study groups.

At the onset of the study (H0), PCV and PVV animals were ventilated with identical mean V_*T*_ [6.90 ± 0.78 (mean ± SD) *versus* 6.67 ± 0.44 mL/kg in PCV and PVV, respectively]. Despite a constant P_*driving*_ throughout the 6-h ventilation, there was a significant and progressive reduction in mean V_*T*_ in both experimental groups, starting from H1 in the PCV group (*p* < 0.001) and from H2 in the PVV group (*p* < 0.001). Notably, after 6 h of ventilation, mean V_*T*_ was significantly lower in animals ventilated with PCV (*p* < 0.05). To target normocapnia, a significant increase in RR was necessary in both experimental groups, in comparison to H0, with no evidence for a statistical difference in RR between the experimental groups. No evidence for intrinsic PEEP or air trapping was observed during the experimental protocol, as assessed though continuous monitoring of expiratory pressure and flow.

The analysis of the ventilatory pattern revealed differences between the experimental groups (Table 1). While animals in the PCV group received nearly constant V_*T*_ and RR, those in the PVV group received a variable pattern for V_*T*_ and RR with a comparable coefficient of variation of approximately 13%.

### Gas Exchange

**FIGURE 4:**
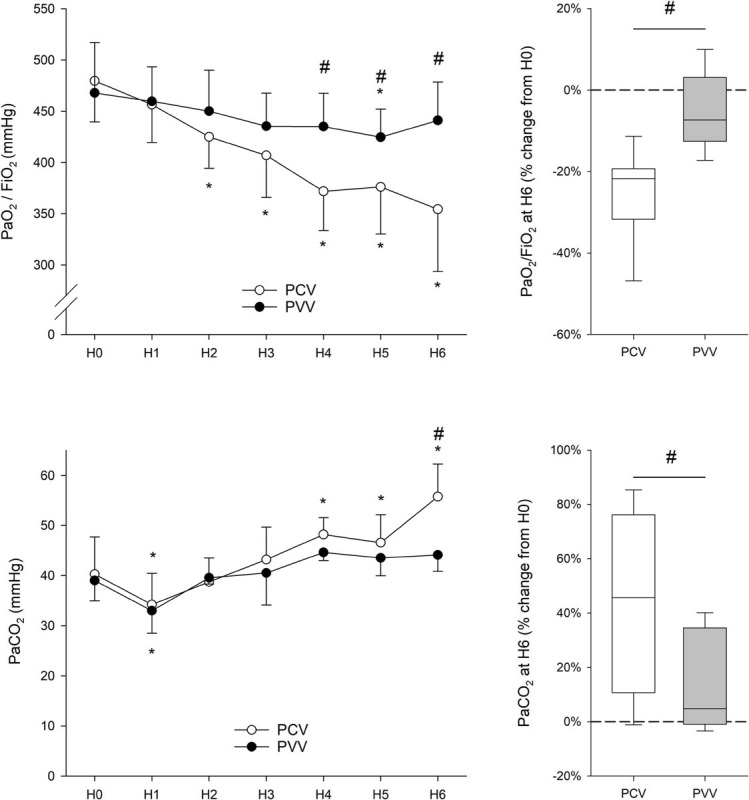
Gas exchange parameters expressed as oxygenation index (PaO_2_/FiO_2_) and partial pressure of CO_2_ in the arterial blood (PaCO_2_), measured at the onset (H0) and throughout the 6 h of ventilation (H1 to H6). Results from physiologically variable ventilation (PVV, filled circles) and pressure-controlled ventilation (PCV, empty circles) are expressed as mean ± standard deviation. Relative changes compared to H0 after the application of 6-h PCV (white box) or PVV (gray box) are reported on the right panels. PaO_2_, arterial partial pressure of oxygen; FiO_2_, fraction of inspired oxygen. *p < 0.05 *versus* H0; #*p* < 0.05 *versus* PCV.

Changes in PaO_2_/FiO_2_ and PaCO_2_ during the study protocol are summarized in Figure 4. Despite COPD induction, O_2_ and CO_2_ levels at H0 were in the physiological range in both groups. While PaO_2_/FiO_2_ remained constant throughout the 6-h ventilation with PVV, it progressively decreased under PCV, and this decrease became significant after 2 h of ventilation (*p* < 0.001). Subsequently, animals in the PVV group exhibited significantly higher PaO_2_/FiO_2_ in the second half of the ventilation period (*p* < 0.001).

Despite a lack of difference in RR between the study groups (Figure 3), the animals in the PCV group presented a significantly higher PaCO_2_ after 6 h of mechanical ventilation than those in the PVV group (*p* < 0.001).

Mean arterial pressure and heart rate elevated during the 6-hour ventilation with no difference between the protocol groups (Supplementary Figure S1).

### Respiratory Mechanical Parameters

**FIGURE 5:**
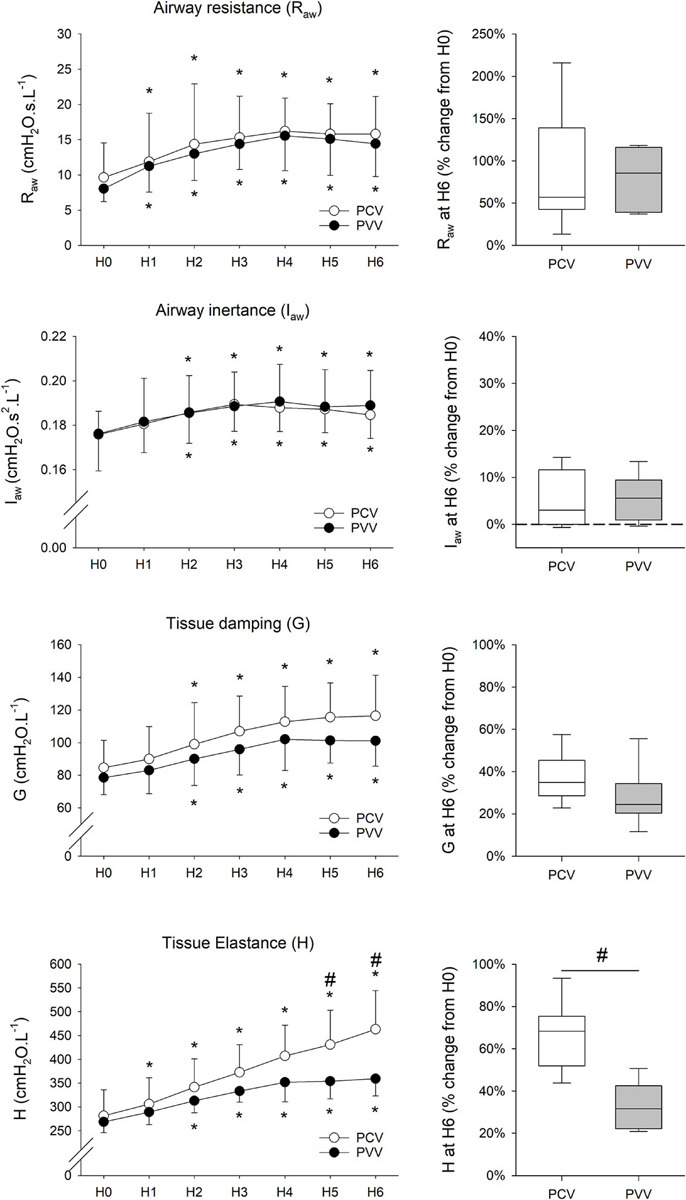
Respiratory mechanical parameters (left panels) in rabbits at the onset (H0) and during 6 h of ventilation (H1 to H6) receiving physiologically variable ventilation (PVV, filled circles) or pressure-controlled ventilation (PCV, empty circles). Values are expressed as mean ± standard deviation. Relative changes compared to H0 after the application of 6-h PCV (white box) or PVV (gray box) are reported in the panels on the right. R_*aw*_, airway resistance; I_*aw*_, airway inertance; G, respiratory tissue damping; H, respiratory tissue elastance. *p < 0.05 *versus* H0; #*p* < 0.05 *versus* PCV.

**FIGURE 6:**
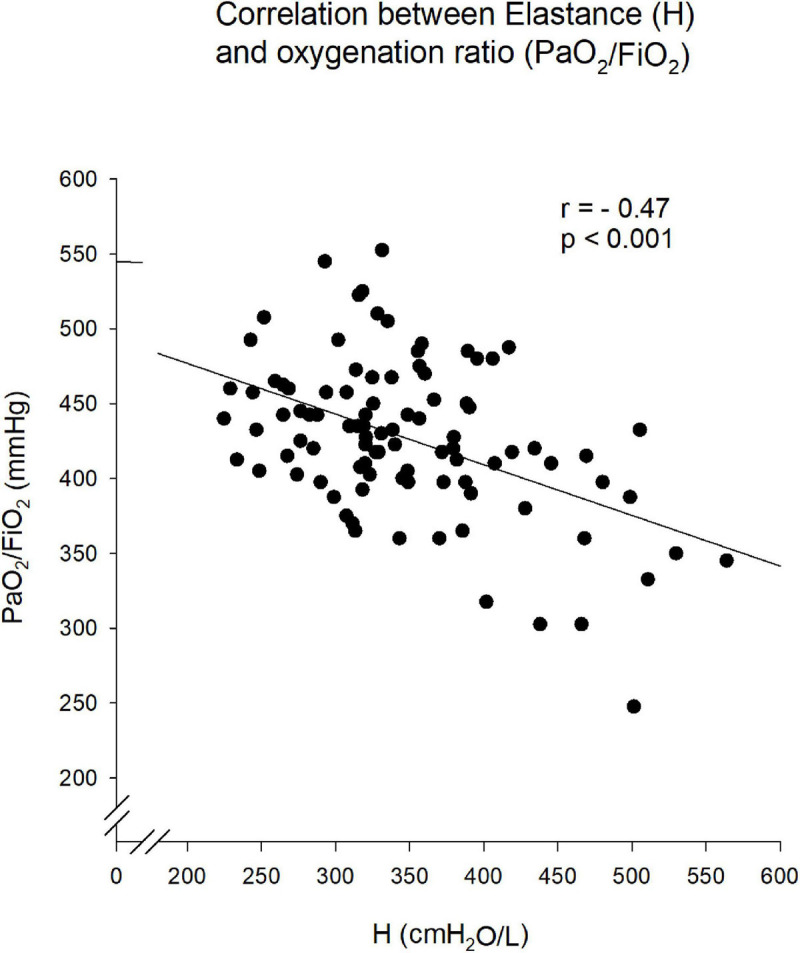
Relationship between the tissue mechanical parameter elastance (H) and the oxygenation index (calculated as partial pressure of oxygen in the arterial blood divided by the fraction of inspired oxygen, PaO_2_/FiO_2_). The trend line corresponds to the best linear fit. Pearson’s correlation coefficient (*r*) shows statistical significance (*p* < 0.001).

All mechanical parameters (R_*aw*_, I_*aw*_, G, and H) progressively increased after 6 h of mechanical ventilation, irrespective of the ventilation mode (Figure 5). Nevertheless, increases in H were significantly lower after 6 h of mechanical ventilation with PVV compared to PCV (*p* = 0.002). Moreover, there was a significant correlation between H and oxygenation index during the 6 h of ventilation (*r* = –0.47, *p* < 0.001, Figure 6).

### Lung Aeration Area and Intrapulmonary Shunt Fraction

**FIGURE 7:**
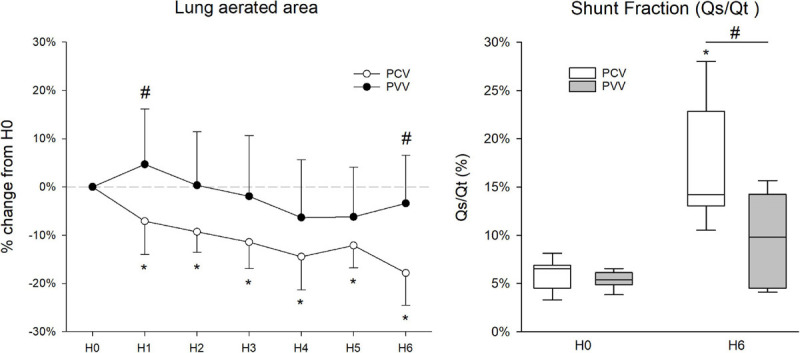
Percentage change of lung aerated area (left graph) obtained from chest X-ray imaging during the 6 h of ventilation (H1 to H6) relative to the lung aerated area at the ventilation onset (H0). Values are expressed as mean ± standard deviation. On the right, shunt fraction (Qs/Qt, in percentage) before and after 6 h of mechanical ventilation (H0 and H6, respectively). Results from physiologically variable ventilation (PVV) and pressure-controlled ventilation (PCV) are represented with filled and empty circles/boxes, respectively. *p < 0.05 *versus* H0; #*p* < 0.05 *versus* PCV.

The relative change in lung aeration area during the 6-h ventilation period is represented in Figure 7. While lung aeration remained unchanged during the study period in the PVV group, a significant and progressive deterioration in lung aeration appeared from the first hour of ventilation in the PCV group (*p* = 0.02). This decrease in lung aeration area became significant between the two groups at H6 (*p* = 0.007).

No difference in Qs/Qt was observed at H0 between the protocol groups; however, intrapulmonary shunt fraction was significantly elevated at H6 only in animals ventilated with PCV (*p* < 0.001). This increase resulted in significantly higher Qs/Qt in PCV compared to PVV at H6 (*p* = 0.002).

### Histological, Cellular, and Protein Assessment

**FIGURE 8:**
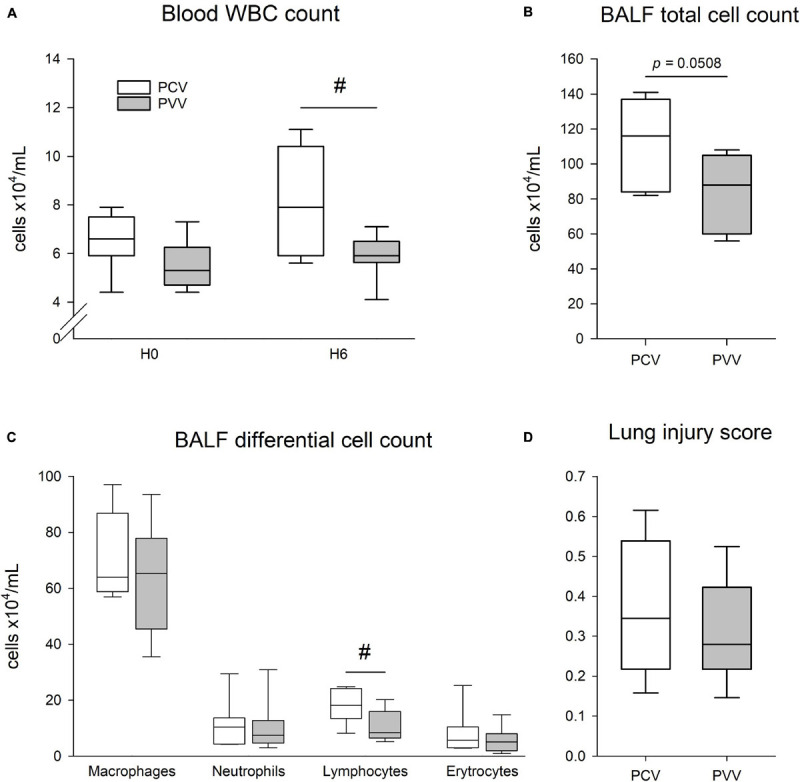
**(A)** Total white blood cell (WBC) count in the blood at the onset (H0) and at the end of the 6-h period of mechanical ventilation (H6) with pressure-controlled (PCV, white) or physiologically variable modes (PVV, gray). **(B,C)** Total and differential cell counts in the bronchoalveolar lavage fluid (BALF) after 6 h of mechanical ventilation with PCV and PVV. **(D)** Lung injury score (range: 0–1) assessed in the lung histological sections after application of 6 h of PVV or PCV. Data are represented as median and quartiles #*p* < 0.05 *versus* PCV.

**FIGURE 9:**
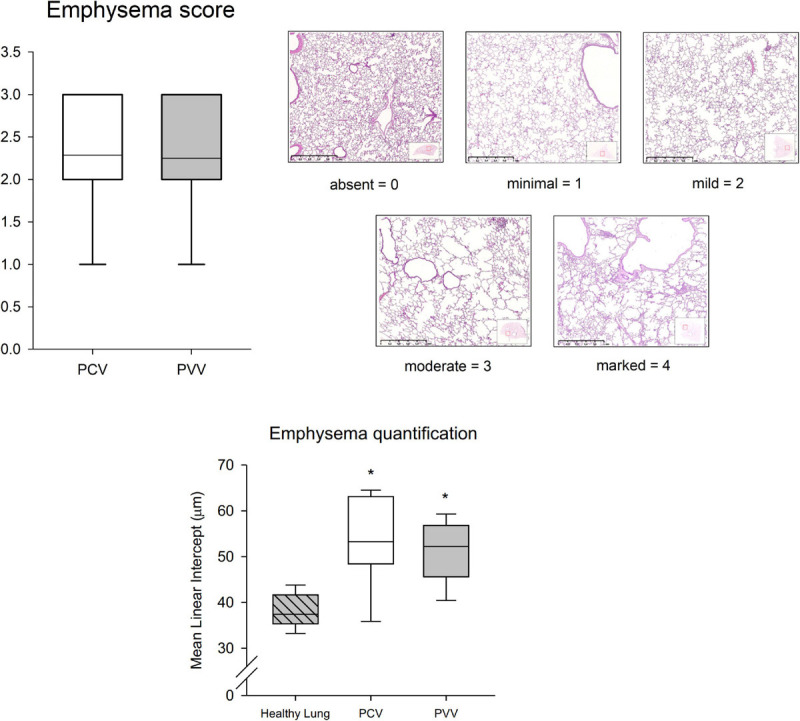
Emphysema pathology score (upper graph), using a five-tier system with 0–4 grading scale: 0, absent; 1, minimal; 2, mild; 3, moderate; and 4, marked. Classification was based on the most severe lesions in 18 lung step slices per animal, anterior to posterior. Example micrographs of each score category are represented in the upper right panels. In the lower graph, airspace enlargement is quantified in histological sections of healthy lungs from control rabbits and PCV and PVV rabbits using MLI (μm). Data are represented as median and quartiles for PCV (pressure-controlled ventilation) and PVV (physiological variable ventilation). *p < 0.05 *versus* healthy lung.

Pathological findings are summarized in Figure 8. Although no difference in the WBC count at H0 was observed between groups, a significant increase was detected at H6 in the PCV group (*p* = 0.04). In addition, there was a tendency for a significant increase in the total cell count in the BALF in the PCV group in comparison to PVV (*p* = 0.051). Moreover, the differential cell count in the BALF revealed a significantly higher number of lymphocytes in the PCV group (*p* = 0.02). Proteins and cytokine concentrations in the BALF and in the lung tissue revealed no significant difference between the two groups (Table 2). After application of PCV and PVV, there was no evidence for a difference in the overall histological injury score (Figure 8D) or in its five histological components (neutrophils in the alveolar and interstitial spaces, hyaline membranes, proteinaceous debris, and alveolar septal thickening) (Supplementary Figure S2). Of note, both groups of animals presented similar degrees of lung tissue inflammation and emphysema, as assessed with MLI and pathology scoring system (Figure 9).

**TABLE 2:** Protein concentration in the supernatant of the bronchoalveolar lavage fluid (BALF), and in frozen lung tissue homogenate are expressed as mean ± standard deviation for pressure-controlled ventilation (PCV) and physiological variable ventilation (PVV).

**BALF**	**PCV**	**PVV**
Total protein (μg/mL)	0.11 ± 0.03	0.13 ± 0.06
TNF-α (pg/mL)	38.8 ± 7.88	41.04 ± 15.88
IL-8 (pg/mL)	238.66 ± 96.68	250.80 ± 82.42
IL-6 (pg/mL)	2.60 ± 2.35	3.79 ± 3.18
SP-B (ng/mL)	0.32 ± 0.37	0.31 ± 0.20
SP-D (ng/mL)	0.28 ± 0.05	0.44 ± 0.36
E-cadherin (ng/mL)	0.40 ± 0.66	0.58 ± 0.69

**Lung tissue**	**PCV**	**PVV**

TNF-α (pg/mg of protein)	Out of range*	Out of range*
IL-8 (pg/mg of protein)	39.49 ± 14.36	39.65 ± 10.29
IL-6 (pg/mg of protein)	9.03 ± 2.44	7.32 ± 1.66
SP-B (ng/mg of protein)	0.82 ± 0.22	0.76 ± 0.23
SP-D (ng/mg of protein)	2.90 ± 0.73	3.89 ± 1.22
E-cadherin (ng/mg of protein)	2.70 ± 0.70	2.38 ± 0.55

TNF, tumor necrosis factor; IL, interleukin; SP, surfactant protein.*Optical density values were out of the absorbance detection limits, equally in both experimental groups.

## Discussion

In an experimental model of COPD, mechanical ventilation with a physiologically variable mode had a beneficial effect compared to the conventional pressure-controlled mode by preventing deterioration in oxygenation and respiratory mechanics. In addition, PVV, in contrast to PCV, protected from declines in tidal volume, intrapulmonary shunt fraction, and lung aeration over time. While these beneficial effects were also reflected in the cytology findings, no clear difference was observed in histological injury.

The experimental model applied in the present study aimed at reproducing the hallmark features of COPD, including lung inflammation and emphysematous airspace enlargement, associated with peribronchiolar infiltrates, inflammation, and thickening in the interstitial and alveolar spaces (Wittels et al., 1974; Kuhn et al., 1976; Mahadeva and Shapiro, 2002; Vernooy et al., 2002). Indeed, the histological analysis confirmed the presence of emphysema and inflammation (Figure 9 and Supplementary Figure S2, respectively). Moreover, lung pathology revealed heterogeneous inflammatory cell infiltrates in the alveolar, interstitial, and peribronchiolar spaces, and rare appearance of multifocal hemorrhages and hypertrophy of the muscular media of small pulmonary arteries. These pathological changes were obtained by combining elastase and LPS nebulization, as described previously in rodents (Sajjan et al., 2009; Ganesan et al., 2010). In this experimental protocol, we did not reproduce the characteristics of airway obstruction and intrinsic PEEP that are often observed while ventilating COPD patients, especially during acute exacerbations of the disease. Instead, our model recapitulates a stable COPD scenario (i.e., when a COPD subject requires mechanical ventilation during anesthesia). Despite potential differences between experimental models of COPD and the complex pathophysiology of COPD in humans (Wright et al., 2008), the model applied in the present study presented the main histological features of COPD. Therefore, it can be considered as a reliable model to assess the potential deleterious effects of mechanical ventilation in the presence of COPD.

The variable ventilation pattern applied in the present study was recorded in awake rabbits after induction of COPD. We hypothesized that the breathing pattern of healthy subjects would not be adequate for chronically diseased lungs, namely, in this model of COPD. This approach contrasts with previous applications of variable ventilation where the pattern was generated from healthy, spontaneously breathing animals (Lefevre et al., 1996; Walesa et al., 2018) or with random variability (Arold et al., 2002). This difference may explain the lower coefficient of variation obtained in the present study compared to that reported for healthy rabbits (Walesa et al., 2018). This finding is in agreement with available data demonstrating decreased breathing variability in the presence of COPD and restrictive lung diseases (Brack et al., 2002; Huhle et al., 2016). Therefore, we find it a reasonable assumption that the variable ventilation pattern applied in the present study resembles the spontaneous breathing observed in COPD subjects.

One major finding of the present study was the improved gas exchange with PVV compared to conventional PCV. Despite equal mean P_*driving*_ and RR between the two ventilation modes, prolonged application of PVV prevented the deterioration of both oxygenation and PaCO_2_. Similar benefits of variable ventilation on gas exchange were reported for models of prolonged ventilation of healthy lungs (Mutch et al., 2000a), atelectasis (Mutch et al., 2000b; McMullen et al., 2006), prematurity (Bartolak-Suki et al., 2017), bronchospasm (Mutch et al., 2007), and acute lung injury with nebulized LPS (Arold et al., 2002). Concerning CO_2_ clearance, animals in the PVV group remained normocapnic throughout the 6-h ventilation period, while those in the PCV group developed progressive hypercapnia. This phenomenon was observed despite a comparable RR between groups. Further increase in RR would have led to development of air trapping and auto-PEEP, despite the prolonged expiratory time with I:E ratio of 1:3.

Regardless of the ventilation mode, the deterioration observed in all respiratory mechanical parameters after mechanical ventilation in supine position can be attributed to progressive lung derecruitment. Since respiratory elastance reflects lung volume loss due to peripheral airway closure (Lutchen et al., 1996; Albu et al., 2013), the limited deterioration in H observed in animals with PVV suggest that application of variable ventilation prevented this alveolar derecruitment. These findings in an experimental model of COPD are in accordance with previous reports in other experimental conditions (Lefevre et al., 1996; Mutch et al., 2000b; Arold et al., 2002; Walesa et al., 2018), where variable ventilation improved lung distensibility (H or respiratory compliance). Moreover, the significant correlation obtained between H and PaO_2_/FiO_2_ further confirms that the increase in H can be attributed to the loss of alveolar units available for gas exchange. The ability of PVV to protect from the deterioration of respiratory elastance was associated with maintenance of lung aeration and a less severe decline in mean V_*T*_, which further support the recruitment effect of PVV in the presence of COPD. This recruitability effect observed for variable ventilation agrees with previous results reported in the presence of ARDS (Graham et al., 2011; Ruth Graham et al., 2011; Huhle et al., 2016).

Another beneficial effect of PVV was manifested in the ameliorated ventilation-perfusion matching that was estimated from the intrapulmonary shunt fraction. The Qs/Qt was in the physiological range for both groups of animals at onset of the ventilation period (Marino, 2014). While intrapulmonary shunt fraction increased after the 6-h ventilation with PCV, physiological ventilation-perfusion matching was preserved in the animals under PVV (Figure 7). This finding is in accordance with the improved aeration, lower derecruitment, and maintenance of normocapnia observed in animals ventilated with PVV.

Assessing the effect of PVV on inflammatory cells revealed benefits both in the blood and the BALF. The effects of variable ventilation on lung inflammatory response are a subject of controversy (Huhle et al., 2016). Our results obtained with the COPD model support earlier observations on the benefit of variable ventilation on lung inflammation (Boker et al., 2002; Arold et al., 2003; Kiss et al., 2016). It is worth noting that all the beneficial effects of PVV outlined above were not reflected in the cytokine profiles and lung histological findings. This result can be attributed to the already established lesions induced by LPS and elastase in the lungs, which likely produce more profound injury than that produced by ventilation. Accordingly, the basal cell counts in the BALF and the histological scores measured in the present model of COPD were correspondingly higher and more severe than those reported earlier for healthy rabbits ventilated with PVV or PCV for 7 h (Walesa et al., 2018).

Variable ventilation has been previously studied in a rat model of elastase-induced emphysema (Henriques et al., 2016), using mathematically random variability over the course of 2 h. This study reported a benefit in lung elastance but failed to observe any effect in gas exchange. While the timespan of the experimental ventilation might have been too short to detect effects in oxygenation, also the pattern and extent of variability might not be appropriate to emphysematous lungs. In fact, it has been demonstrated that too much variability can have deleterious effects (Nam et al., 2000; Wierzchon et al., 2017). On the contrary, in the present study, the driving signal of variable ventilation was the pattern of spontaneous breathing recorded in awake rabbits, after COPD features were induced by a combination of elastase and LPS.

This study has a certain number of limitations which warrant consideration. First, a smaller number of animals was included in the 6-h ventilation period that the estimated sample size. Despite the optimization of the COPD induction protocol, one-third of the experimental animals did not survive the elastase nebulization. However, evidence for the benefits of PVV over PCV was already demonstrated with smaller number of animals and thus, requesting additional animals to reach the initially estimated sample size could not be justified in view of the recommendations on the reduction of the use of animals in research (Russell and Burch, 1959). Second, sham-treated rabbits were not included in the present study. Instead, the current study outcomes were compared to previous results obtained in ventilated healthy rabbits from our research group (Walesa et al., 2018). Since these previous experiments were performed under identical conditions, the comparisons are valid and compliant with the recommendations on the reduction of the use of animals in research (Russell and Burch, 1959), as requested by the local animal welfare committee. Third, the timespan of the mandatory ventilation in the present study was limited to 6 h. Thus, extrapolating the present results for longer term ventilation and outcomes may be limited. Of note, a recent clinical trial failed to demonstrate long-lasting benefits when applying a mathematical model of variable ventilation in healthy lungs (Spieth et al., 2018). Finally, the design and sample size calculation of the study were not powered to demonstrate histological and cytokine outcomes.

## Conclusion

In summary, the comparison of PVV to conventional PCV in an experimental model of COPD revealed that the introduction of physiological variability to mechanical ventilation improves oxygenation, CO_2_ clearance, respiratory tissue mechanics, tidal volume, lung aeration, and intrapulmonary shunt fraction. Thus, in a model of COPD, PVV has the ability to prevent alveolar derecruitment, thereby reducing alveolar shear stress with subsequent improvement in gas exchange. Therefore, our results encourage the consideration of PVV as a protective ventilation modality in the context of COPD.

## Data Availability Statement

The raw data supporting the conclusions of this article will be made available by the authors, without undue reservation.

## Ethics Statement

The animal study was reviewed and approved by the Animal Welfare Committee of the Canton of Geneva and the Experimental Ethics Committee of the University of Geneva, Switzerland (GE 184/18, 2 January 2019).

## Author Contributions

ADSR, RS, RD, FP, and WH contributed to study design. ADSR, RS, and DB contributed to experimental work. ADSR, RS, DB, MK, TC, FP, and WH contributed to data analyses. ADSR, FP, and WH contributed to manuscript drafting. ADSR, RS, DB, MK, TC, RD, FP, and WH contributed to manuscript review and editing. All authors read and approved the final manuscript.

## Conflict of Interest

The authors declare that the research was conducted in the absence of any commercial or financial relationships that could be construed as a potential conflict of interest.
