# Supplementary material for: Benefit of Physiologically Variable Over Pressure-Controlled Ventilation in a Model of Chronic Obstructive Pulmonary Disease: A Randomized Study
Source: Front Physiol. 2021 Jan 13;11:625777. doi: 10.3389/fphys.2020.625777 (PMC7839245; doi:10.3389/fphys.2020.625777)
Supplement: Supplementary file 1 [file Table_1.DOCX]

Supplementary Material

Benefit of physiologically variable over pressure-controlled ventilation in a model of chronic obstructive pulmonary disease: a randomized study

**Andre Dos Santos Rocha ^1*^, Roberta Südy ^1^, Davide Bizzotto ^2^, Miklos Kassai ^1^, Tania Carvalho ^3^, Rafaelle Dellacà ^2^, Ferenc Peták ^4^, Walid Habre ^1^**

^1^ Unit for Anaesthesiological Investigations, Department of Acute Medicine, University Hospitals of Geneva and University of Geneva, Geneva, Switzerland

^2^ Dipartimento di Elettronica, Informazione e Bioingegneria, Politecnico di Milano, Milan, Italy

^3^ Instituto de Medicina Molecular, Faculdade de Medicina, Universidade de Lisboa, Lisbon, Portugal

^4^ Department of Medical Physics and Informatics, University of Szeged, Szeged, Hungary

*** Correspondence:**Andre DOS SANTOS ROCHA
[Andre.DosSantosRocha@unige.ch](mailto:Andre.DosSantosRocha@unige.ch)

Unit for Anaesthesiological Investigations, Dept of Acute Medicine, University of Geneva

Rue Michel-Servet 1, 1206 Genève, Switzerland

Ph: +41 (0)79 553 21 36, e-mail: Andre.DosSantosRocha@unige.ch

**Figure S1**

**Figure S1**. Hemodynamic parameters obtained during the 6-hour ventilation period. Values expressed as mean ± standard deviation. MAP: mean arterial pressure; HR: heart rate; H0 to H6: average value during the corresponding hour of the 6-hour long ventilation period; PCV: pressure-controlled ventilation; PVV: physiological variable ventilation. *: p < 0.05 vs. H0

**Figure S2**


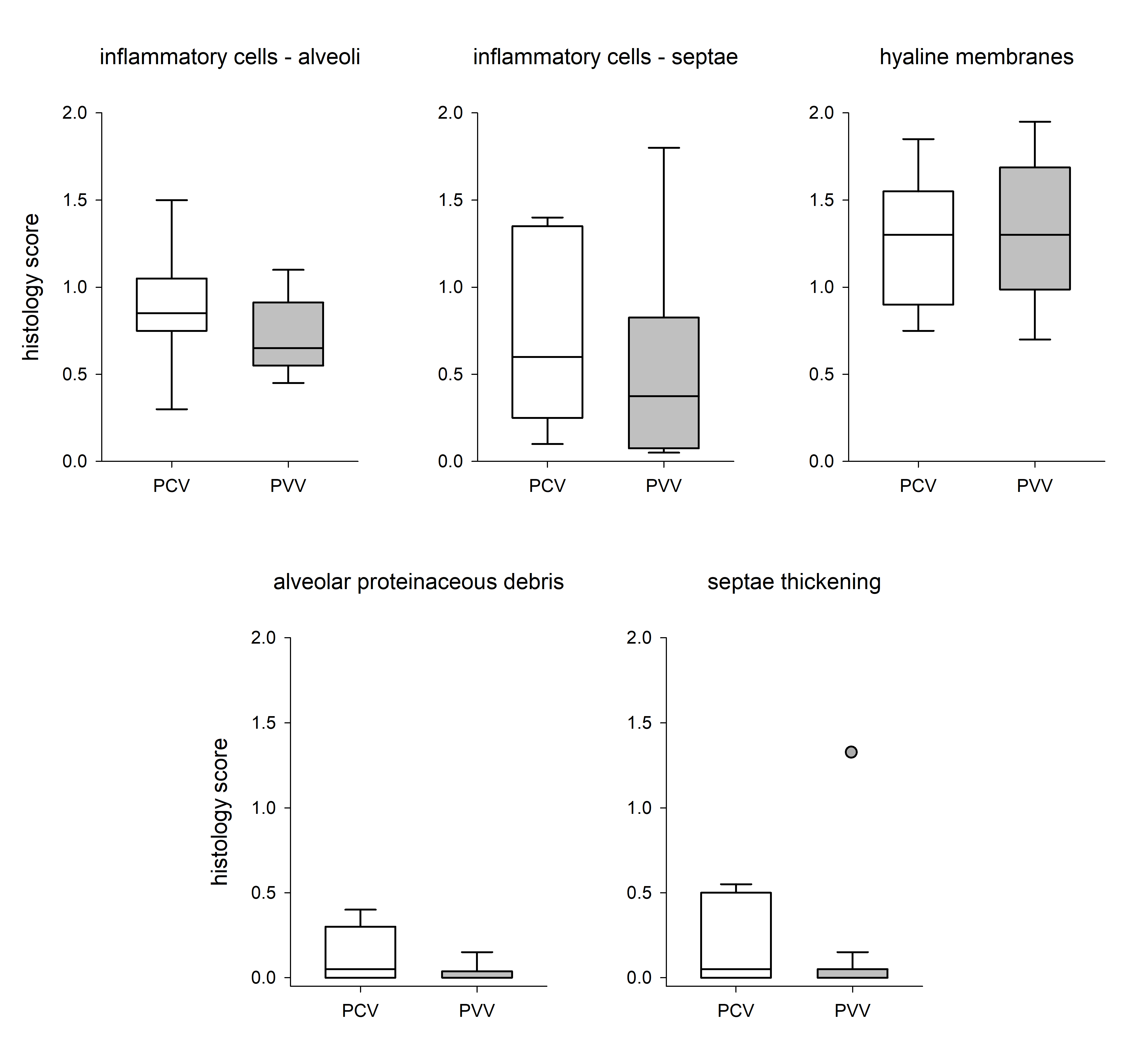


**Figure S2**. Quantification of the five histological components of the Lung Injury Scoring System, summarized in Figure 6, using the guidelines of the American Thoracic Society (7). Accordingly, a score per field (between 0 and 2) was averaged from 18 lung step slices, anterior to posterior, of the left lung. Data in the box plot is represented as median and quartiles. PCV: pressure-controlled ventilation; PVV: physiological variable ventilation.

**Table S1.** Animal welfare score, approved by the Animal Welfare Committee of the Canton of Geneva and the Experimental Ethics Committee of the University of Geneva, Switzerland (GE 184/18, January 2^nd^, 2019), for bi-weekly assessment of rabbit well-being during COPD induction. If the total score was ≥1, rabbits would receive supplementary oxygen. BW: body weight.

| **Animal Welfare Score (Protocol GE 184/18)**  For each parameter, attribute a score of 0 (absent) or 1 (present). |  |
| --- | --- |
| Reduced activity | |
| No grooming | |
| Pale eyes | |
| Folding of the eyelids | |
| Reduction of food/water intake | |
| Weight loss (> 5% BW between 2 evaluations or 10% in one week) | |
| Change of posture, folding of the abdomen, muscle tension | |
| On guard, tendency to hide or be aggressive | |
| Dyspnea (noisy breathing, sneezing, respiratory rate >60/min) | |
